# A novel yeast-based high-throughput method for the identification of protein palmitoylation inhibitors

**DOI:** 10.1098/rsob.200415

**Published:** 2021-08-04

**Authors:** Consuelo Coronel Arrechea, María Luz Giolito, Iris Alejandra García, Gastón Soria, Javier Valdez Taubas

**Affiliations:** ^1^ Centro de Investigaciones en Química Biológica de Córdoba (CIQUIBIC) CONICET, Córdoba, Argentina; ^2^ Departamento de Química Biológica Ranwel Caputto, Facultad de Ciencias Químicas, Córdoba, Argentina; ^3^ Departamento de Bioquímica Clínica, Facultad de Ciencias Químicas, Universidad Nacional de Córdoba, Córdoba, Argentina; ^4^ Centro de Investigaciones en Bioquímica Clínica e Inmunología, CIBICI-CONICET, Córdoba, Argentina

**Keywords:** S-acylation, protein palmitoylation, drug discovery, inhibitors, yeast

## Abstract

Protein S-acylation or palmitoylation is a widespread post-translational modification that consists of the addition of a lipid molecule to cysteine residues of proteins through a thioester bond. Palmitoylation and palmitoyltransferases (PATs) have been linked to several types of cancers, diseases of the central nervous system and many infectious diseases where pathogens use the host cell machinery to palmitoylate their effectors. Despite the central importance of palmitoylation in cell physiology and disease, progress in the field has been hampered by the lack of potent-specific inhibitors of palmitoylation in general, and of individual PATs in particular. Herein, we present a yeast-based method for the high-throughput identification of small molecules that inhibit protein palmitoylation. The system is based on a reporter gene that responds to the acylation status of a palmitoylation substrate fused to a transcription factor. The method can be applied to heterologous PATs such as human DHHC20, mouse DHHC21 and also a PAT from the parasite *Giardia lamblia*. As a proof-of-principle, we screened for molecules that inhibit the palmitoylation of Yck2, a substrate of the yeast PAT Akr1. We tested 3200 compounds and were able to identify a candidate molecule, supporting the validity of our method.

## Graphical abstract

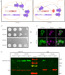

## 1. Introduction

Numerous proteins are post-translationally modified by the addition of a lipid molecule on the amino acid cysteine, through a thioester bond. This modification, known as palmitoylation, is best described as S-acylation since the length of the lipid chains that are incorporated is variable (reviewed in [1]). Protein S-acylation is a reversible lipid modification, and therefore capable of exerting regulatory roles [2–4]. Some peripheral proteins such as Ras or G proteins are palmitoylated, often in combination with prenylation, resulting in their recruitment to membranes. Proteins with transmembrane domains (TMDs) can also be palmitoylated [5]. S-acylation is one of the most prevalent post-translational modifications. In humans, over 12% of the proteome is thought to be S-acylated [6]. The role of palmitoylation is increasingly recognized in processes such as the visual cycle, signal transduction, synaptic transmission [1,7–10] and protein trafficking (reviewed in [11]).

A family of proteins characterized by the presence of a 50 amino acid domain, rich in cysteines, called DHHC-CRD (Asp−His−His−Cys−Cysteine-Rich Domain), is responsible for S-acylation (reviewed in [12,13]). These proteins were initially identified in the yeast *Saccharomyces cerevisiae,* which has seven members of this family encoded in its genome. The DHHC family proteins, or palmitoyltransferases (PATs), are polytopic membrane proteins that have at least four TMDs, with the DHHC domain located between TMDs 2 and 3. There are 23 members of this family in the human genome [1,12,13].

The mechanism of S-acylation has been elucidated and consists of two stages. In the first stage, the PATs auto-acylate in the conserved cysteine of the DHHC motif using acyl-CoA as a donor substrate, and in a second stage, they transfer the palmitate to the acceptor cysteine/s of the substrate [14,15], however, it has been shown that for certain enzyme–substrate pairs, palmitoylation might occur by a non-canonical route [16].

Mutations in PATs have been associated with mental retardation, schizophrenia and other disorders of the central nervous system [17,18]. Palmitoylation of some protozoan parasite proteins is essential for invasion of host cells (reviewed in [19,20]) and some bacteria inject effectors that must be palmitoylated by the host machinery to be active and promote infection [21–24]. S-acylation of proteins has been involved in numerous types of cancer, mainly due to its action on proteins involved in signal transduction cascades. Some PATs have been described as oncoproteins and others as tumour suppressors (reviewed in [25,26]). For instance, DHHC21 and DHHC7 are involved in the palmitoylation of steroid hormones receptors α (oestrogen, progesterone and androgens) [27]. These receptors, while exercising many of their functions at the nuclear level, also have activity outside the nucleus. Aggressive, or resistant to endocrine therapy breast cancer, is sometimes associated with a significant increase in the location and function of oestrogen α receptors outside the nucleus [28]. Searches in the Oncomine database revealed that the DHHC21 gene is significantly overexpressed in human breast cancer [27]. Therefore, DHHC21 and DHHC7 PATs could be targeted for the treatment of breast cancer in which steroid hormone receptors are required for cell proliferation and survival [27]. It is interesting that DHHC7 and DHHC21 also modify the amyloid precursor protein [28]. It has been proposed that strategies aimed at the prevention of amyloid precursor protein (APP) palmitoylation through the development of specific inhibitors could prevent and/or treat Alzheimer's disease (reviewed in [29]).

Of the many studies that suggest the use of PATs inhibitors as possible therapeutic agents, perhaps one of the most interesting is the one involving the palmitoylation of the epidermal growth factor receptor [30] mediated by DHHC20. The elimination of this modification by silencing DHHC20 with small interfering RNA sensitizes the cells to inhibitors of receptors' tyrosine kinase activity, such as Gefitinib [31], which is currently used as a therapeutic agent in some types of breast and lung cancer [32]. Research from the same group has recently shown that blocking estimated glomerular filtration rate palmitoylation by knocking down DHHC20 reduced tumour growth in a mouse model of KRAS-mutant lung adenocarcinoma and increased the sensitivity of these cells to a PI3 K inhibitor [33].

For all the above, there is great interest in identifying specific S-acylation inhibitors [1,34,35]. Currently, the palmitoylation inhibitor most commonly used in experimentation is 2-bromopalmitate (2-BP) which, in addition to inhibiting palmitoylation in general, can under certain conditions inhibit de-palmitoylation [36]. It is also known that this compound inhibits a wide variety of lipid-metabolism enzymes [37]. This tool must be used with extreme caution and it is urgent to identify specific inhibitors to advance knowledge of this field.

A screen on chemical compound collections has been performed in search of S-acylation inhibitors [38]. However, the reported potency and selectivity of these compounds were not recapitulated with purified enzymes and their *bona fide* substrates [39]. A yeast-based assay allowed the identification of an inhibitor of an endogenous PAT (Erf2) by an *in vitro* assay [40]. Recently, in a screen for molecules that inhibit the traffic of the HIV receptor CCR5 to the plasma membrane, two drugs that inhibit the palmitoylation of this receptor were found [41]. These drugs are CdCl_2_ and Zinc pyrithione, which do not appear to be therapeutically viable alternatives given their high toxicity. It was also described that the antifungal Ketoconazole inhibits the palmitoylation of dual leucine-zipper kinase, by an unknown mechanism [42].

Here we report a strategy for the identification of palmitoylation inhibitors, which is based on yeast strains that bear a reporter gene under the transcriptional control of the artificial transcription factor Tf (LexA–VP16) [43]. In turn, the transcription factor in this strain is fused to a palmitoylation substrate. The palmitoylation of this chimaera induces its anchoring to the plasma membrane so that the transcription factor cannot enter the nucleus, resulting in a strain unable to grow in a medium without histidine. Therefore, inhibition of palmitoylation of this chimaera should allow growth in the absence of histidine.

We show that the system can be scaled to a high-throughput format and also that it can be adapted for the identification of PATs of human and parasite origin. Finally, we carried out a proof-of-concept screen for inhibitors of the palmitoylation of the Akr1 substrate Yck2 and identified a candidate molecule, whose specificity and mechanism of action remains to be elucidated.

## 2. Results

### 2.1. Generation of a screening method to identify palmitoylation inhibitors *in vivo*

The screening system is based on the palmitoylation of a PAT substrate fused to a transcription factor that controls a reporter gene. We have chosen as substrate the yeast protein Yck2 which is palmitoylated by the PAT Akr1 and this is necessary for its function. Fusions to this substrate were similarly employed in the search for novel yeast depalmitoylases [44]. Akr1 was one of the firsts PATs to be identified [45]. It is characterized by the presence of Ankyrin repeats in the N-terminal domain, and its most studied substrate is Yck2 (Casein Kinase 1). This plasma membrane kinase has multiple functions in the yeast cell, including morphogenesis, septal assembly, endocytic traffic and glucose detection [46–48]. The palmitoylation of Yck2 by Akr1 in two C-terminal cysteines is necessary and sufficient for its membrane localization [49]. Loss of Akr1 leads to multiple phenotypes including poor growth at 30°C [50].

**Figure 1.**
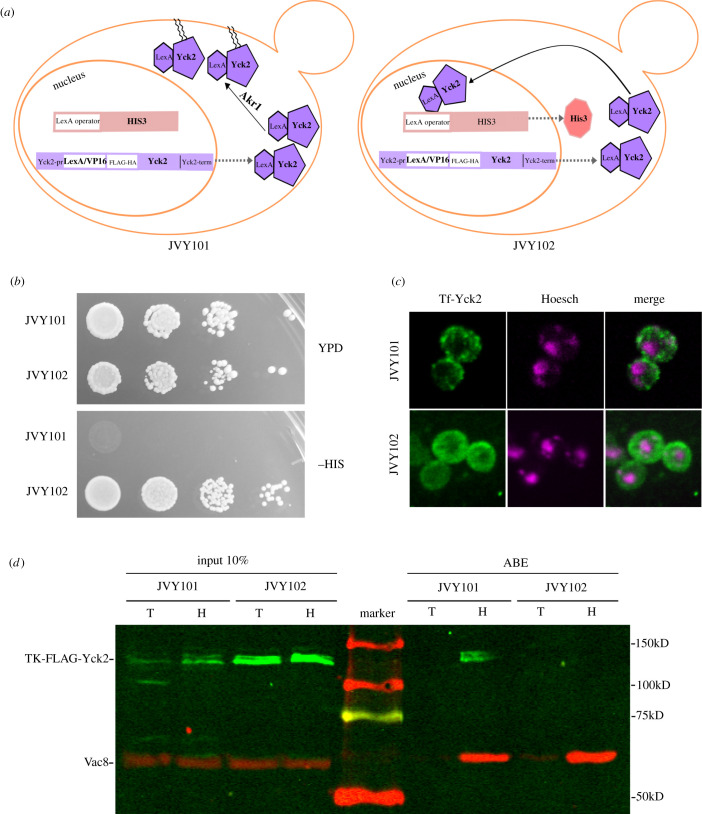
(*a*) Schematics for the strains JVY101 and JVY102. In JVY101 the PAT Akr1 is present, and therefore the chimaera between LexA-Yck2 becomes palmitoylated and attached to the membrane. In JVY102 AKR1 is absent and the chimaera is free to enter the nucleus, where it drives the expression of the HIS3 gene. (*b*) Serial dilutions of JVY101 and JVY102 strains were placed in complete media which contains histidine (YPD) upper panel or in SC media lacking histidine (lower panel). (*c*) The subcellular localization of Tf-Yck2 was established by indirect IF and confocal microscopy, in the JVY101 (upper panels) and JVY102 strains (lower panels). Cells were also labelled with the Hoesch stain to determine the localization of the cells' nuclei. (*d*) S-acylation of Tf-Yck2 in JVY101 and JVY102 strains is assessed by ABE. A positive signal in the hydroxylamine (H) treated samples indicates S-acylation. Additional samples were treated with 1 M Tris pH 7,4 buffer as a negative control. As a positive control for the ABE, the blots were probed using a rabbit polyclonal antibody against the palmitoylated protein Vac8 and a secondary antibody coupled to IRDye680 (red). Tf-Yck2 was developed using an anti-HA monoclonal antibody and a secondary antibody coupled to IRDye 800 (green). Blots are representative of two independent experiments.

We started from the strains NDY1949 and NDY1953, which is NYD1949 with AKR1 gene deleted (kindly provided by Dr Nicholas Davis, Department of Pharmacology, School of Medicine, Wayne State University). These strains have the HIS3 gene under the control of the LexA operator and also express a palmitoylation-independent allele of Yck2 that allows NYD1953 (*akr1Δ*) to grow well at 30°C [45]. In the genomes of these strains, we integrated a cassette that expresses Casein Kinase 1 (Yck2) fused to the C-terminus of the artificial transcription factor LexA/VP16 [43], from now on Tf. Since Yck2 is a substrate of endogenous Akr1, the chimaera should be anchored to the plasma membrane and the strain should not grow in the absence of histidine. If the AKR1 gene is deleted, the resulting strain should grow without histidine (figure 1*a* shows a scheme). The insertion of this cassette was directed to the ERG6 locus, thus deleting the ERG6 gene, which participates in one of the latest steps of ergosterol biosynthesis. This mutation makes yeasts more sensitive to drugs due to an increase in membrane permeability [51].

The system has the advantage that the addition of 3-aminotriazole (3-AT), a competitive inhibitor of the HIS3 gene product, allows controlling the stringency of the screen.

Figure 1*b* shows a growth test of the resulting strains in a solid selective medium. The strain with *wt* Akr1 (JVY101) is unable to grow without histidine, while the strain that lacks Akr1 (JVY102) grows well in this medium. This large difference in growth suggests that Tf-Yck2 is fully palmitoylated in the presence of Akr1 and also that the HIS3 gene is very tightly controlled by the availability of Tf-Yck2 in the nucleus, indicating that a partial loss of Tf-Yck2 palmitoylation by, for instance, inhibition of a PAT activity, should be readily detectable. We carried out indirect immunofluorescence (IF) to establish the subcellular localization of Tf-Yck2. Since Tf-Yck2 expression is driven by the native Yck2 promoter, the protein levels are not very high, making IF detection difficult, nevertheless, we were able to establish that Tf-Yck2 in JVY101 shows a dotted distribution around the cell periphery. The staining markedly differs from that obtained in JVY102 which is mostly cytosolic (figure 1*c*). We expected to see more nuclear staining in this strain, but it seems sufficient to drive HIS3 gene expression.

Finally, the palmitoylation status of Tf-Yck2 was assessed biochemically by acyl-biotin exchange (ABE). In this method, thioester-linked lipid molecules are released from proteins by neutral hydroxylamine and replaced by biotin. Biotin-labelled proteins are pulled down using streptavidin beads and identified by Western blot. Additional samples are treated with TRIS–HCl as negative controls. Figure 1*d* shows that we can detect Tf-Yck2 in the hydroxylamine treated protein extracts from the JVY101 strain but not from JVY102 which lacks Akr1. We consistently observed lower levels of Tf-Yck2 in the JVY101 strain, as can be seen in the input lanes, suggesting that the palmitoylated chimaera is more unstable.

**Figure 2.**
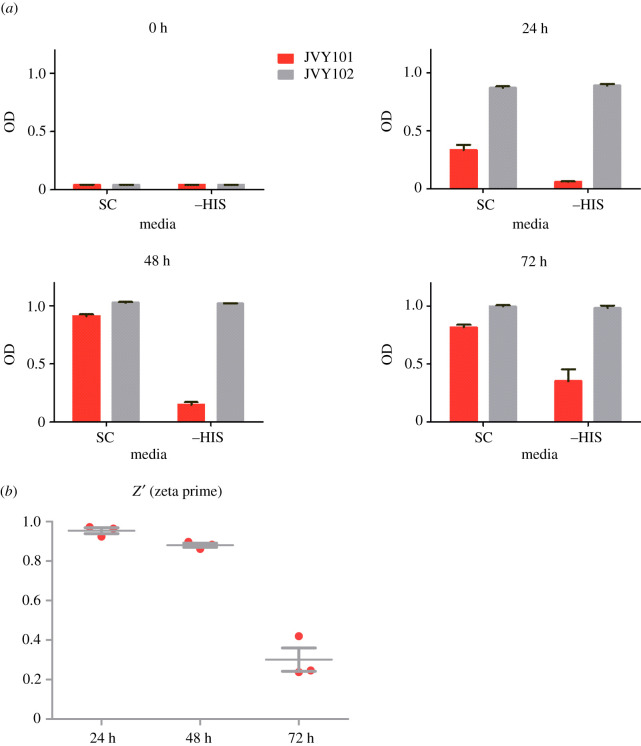
(*a*) Growth test in liquid media using the multiwell format. The graphs show the average value for the optical densities ± standard deviation at 600 nm for the strains JVY101 (red) and JVY102 (grey) at different times post inoculum, from three independent experiments. (*b*) *Z′*values calculated for the screen using JVY101 and JVY102, values above 0.5 are considered excellent. The graph shows the average value for the *Z′* ± standard deviation from three independent experiments.

To test the system in a high-throughput compatible setting, we analysed the behaviour of JVY101 and JVY102 in growth tests in liquid media in 96-well plates. Different amounts of inoculum were tested to find the maximal growth difference between the strains (not shown). Once this was established, growth was assessed by measuring optical density (OD) at 600 nm on multiwell plates at 24, 48 and 72 h on medium with or without histidine. We inoculated 16 wells for each strain and condition. Figure 2*a* shows that at 24 h, JVY101 shows no growth compared to the inoculum, while JVY102 (lacking AKR1) grows to (0.887 ± 0.02) OD in media without histidine (−HIS). The addition of 3-AT did not improve the growth difference between the JVY101 and JVY102 in –HIS, and therefore was omitted from the following assays. At 48hs, the difference between JVY101 and 102 was even higher with a growth of (0.15 ± 0.03) OD for JVY101 and (1.019 ± 0.005) OD for JVY102. At 72 h JVY101 begins to grow, but still to lower levels than JVY102.

To assess if our assay would be useful in a full-scale high-throughput screening, we calculated the *z′* value, which is a statistical parameter that describes the quality of the screens. This parameter has a maximum of 1. Figure 2*b* shows the Zeta prime values, the average value of three different experiments at 24 h is 0.95 ± 0.03 and at 48 h is 0.88 ± 0.02. Screens with *z′* values between 0.5 and 1 are considered excellent [52]. In conclusion, our system robustly discerns between strains with or without Akr1 activity and thus should be useful for the identification of palmitoylation inhibitors.

### 2.2. The screening method can be used with heterologous PATs

One of the phenotypes of yeasts that lack Akr1 is thermo-sensitivity. *akr1Δ* cells cannot grow at 37°C [50]. The bases for this phenotype are not completely understood, but interestingly, it can be complemented by multiple human PATs [53], suggesting that perhaps heterologous PATs could also complement the loss of Akr1 activity in our assay.

We analysed several mammalian PATs, including DHHC21, DHHC20, DHHC6 and DHHC4 and also PATs from the protozoan parasite *Giardia lamblia,* a pathogenic parasite responsible for intestinal infections [54].

Parasite PATs are very interesting drug targets since they are very divergent from mammalian PATs [55] (C.C.A., M.L.G., I.A.G., G.S. & J.V.T. 2009, personal observations). Therefore, a specific inhibitor for a parasitic pathogen may find a way into therapeutics more readily. However, this divergence may also lead to a lack of activity in our assay. First, we tested the complementation of the *akr1Δ* strain thermo-sensitivity by all nine DHHC proteins from *G. lamblia*. The DHHC protein of unknown function gl6733 was shown to complement the loss of Akr1 in this assay, even when expressed from a centromeric vector (electronic supplementary material, figure S1).

**Figure 3.**
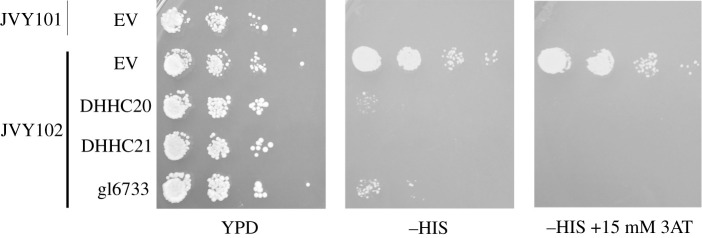
Serial dilutions of JVY101 transformed with an empty vector (EV) and JVY102 transformed with either EV or plasmids expressing DHHC20, DHHC21 and *G. lamblia* gl7733 were placed in complete media (YPD), SC media lacking histidine (−HIS) or SC media lacking histidine with the addition of 15 mM 3-aminotriazole (−HIS + 15 mM 3AT).

To test if heterologous PATs can complement the lack of Akr1 in our assay, complementary DNA (cDNAs) from DHHC6, DHHC4, DHHC20, DHHC21 and gl6733 were cloned in multicopy 2µ expression vectors, under the control of the constitutive TPI1 promoter and transformed into JVY102 (*akr1Δ*). We carried out growth tests in solid media and observed that the expression of either, DHHC20, DHHC21 or gl6733 results in lack of growth in a medium without histidine (figure 3), indicating that Tf-Yck2 is being palmitoylated by these PATs. Some very small growth can be observed on the strains expressing DHHC20 and gl6733, however, the addition of 15 mM 3AT to the medium completely abolishes that residual growth. Strains carrying DHHC4 and DHHC6 plasmids were able to grow in medium without histidine indicating that these plasmids are unable to complement for the lack of AKR1 (not shown).

We next tested if the strain expressing DHHC20 can also perform adequately in a high-throughput set-up. Electronic supplementary material, figure S2, shows that there is a substantial growth difference between JVY102, and JVY102 expressing DHHC20, in the multiwell plates format. The addition of 30 mM 3AT and optimization of the inoculum are required for an optimal growth difference between the strains, confirming that the assay would be useful in the isolation of DHHC20 inhibitors.

**Figure 4.**
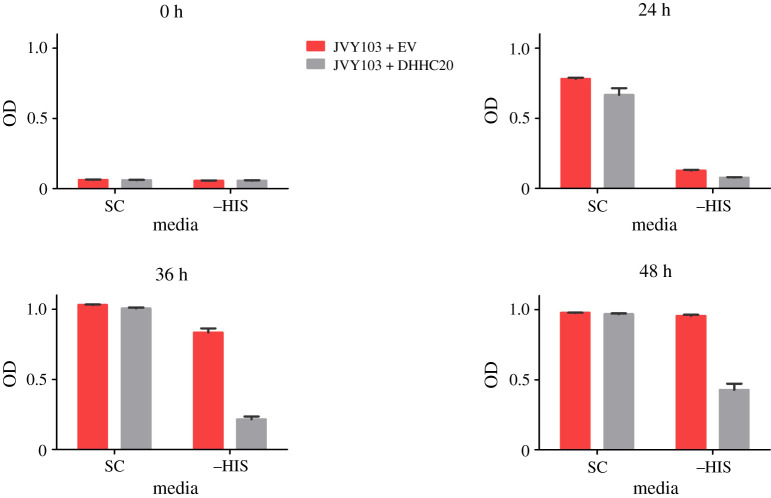
Growth test in liquid media using the multiwell format. The graphs show the average value for the optical densities ± standard deviation at 600 nm for the strains JVY103 (red) which expresses a palmitoylation domain from NCX1.1 transformed with an EV or with a plasmid expressing DHHC20 (grey) at different times post inoculum.

The screening method in principle could be adapted so that instead of evaluating the palmitoylation of Tf-Yck2 by the mammalian PATs, we could look at the palmitoylation of a chimaera between Tf and heterologous *bona fide* substrates of these PATs. This might have the additional advantage of selecting inhibitors that specifically disrupt the palmitoylation of a particular substrate by interfering with the enzyme–substrate interaction. We selected a few protein domains from mammalian proteins that are palmitoylation substrates, fused them to Tf and assessed them with our system. We tried the cytosolic domain of the EGFR, but this was not palmitoylated by endogenous PATs nor by co-expression with DHHC20 (not shown), presumably because the cysteines on this domain have to be close to a membrane to be palmitoylated. In the *wt* EGFR, this occurs by virtue of the TMD, which is absent from our construct. A cytosolic region from IFTM3 [56] was also tested but this strain did not grow in the absence of histidine suggesting that it was efficiently palmitoylated by the remaining yeast PATs in this strain (not shown). Finally, we tested the intracellular loop of NCX1.1, which was shown to be a signal for palmitoylation on adjacent cysteines [57]. This domain was fused to Tf and the construct integrated into JVY102 (*akr1Δ*) genome, to generate JVY103. The strain was able to sustain growth in media without histidine, and growth was partially abolished by the expression of DHHC20 (figure 4), indicating that this domain is being modified by DHHC20.

### 2.3. Proof-of-concept screen

Since the system is based on the palmitoylation of Yck2 by Akr1 and its subsequent anchoring to membranes, it should be optimal for this enzyme–substrate pair. To validate our screening method, we carried out a proof-of-concept screen for inhibitors of Yck2 palmitoylation.

We analysed 3200 compounds, randomly selected from the ‘3D Biodiversity’ library (ChemDiv, CA USA); the compounds present in this library have been selected for their low reactivity and toxicity, as well as for their high structural diversity.

Here 96-well plates with 200 µl of synthetic complete (SC) medium without histidine were inoculated with strain JVY101 at a concentration of 0.005 OD ml^−1^ (600 nm). Subsequently, the drugs were added at a concentration of 10 µM in dimethyl sulfoxide (DMSO). For each plate, DMSO alone is added to eight wells, as negative controls. As a positive control, we used the JVY102 strain (*akr1Δ*). Attempts were made to use also 2-BP as a positive control, but it was toxic at high concentrations (40 µM), and at lower concentrations JVY101 cells fail to grow. OD was monitored at 24, 48 and 72 h.

To help us determine which OD values should be considered as hits, we generated a dot plot for the whole dataset. Since we did not screen all the compounds at once, but rather in batches of around 800 compounds (10 multiwell plates), we chose to plot the data points for each plate. As it can be observed from the graph (electronic supplementary material, figure S3), there are differences in the mean growth for each subset of plates. This allowed us to select some compounds that would not have been selected if we used the mean growth of the complete dataset, plus three s.d. as a criterion for selection, but they stand out when looking at the particular set of plates. Nevertheless, we calculated the mean for the whole dataset and drew a red line in the graph at mean plus 3 s.d. (mean = 0.077, mean + 3 s.d. = 0.201). In total, 24 of the 34 compounds that we selected for further analyses are above this line. However, when analysed within the specific subset of plates, all positives had a growth of mean + 5 s.d. Additionally, we included the values for the positive controls for each plate (blue symbols) and also the mean growth for the JVY102 (grey line) with DMSO. This resulted in a hit rate of 1.06% highlighting the high stringency of our screening method. These 34 compounds were submitted to a first validation round in multiwell plates using 2.5, 5, 10 and 20 µM of each compound (not shown). Four compounds that displayed high values of OD in the primary screen and did not recapitulate this growth in the secondary screen turned out to be bacterial contaminations, and another, a molecule that contained histidine in its structure.

Based on these results, we carried out a third validation, in which the concentration of the compounds was adjusted according to the results of the second validation. In these experiments, we also included a counter screen, in which the compounds were added to a strain that lacks Tf-Yck2 but it is otherwise isogenic to JVY101 (JVY104). These experiments ruled out most of the compounds obtained in the first and secondary screens since they also stimulated the growth of the JVY104 strain in restrictive media (electronic supplementary material, table S2). This third validation was read at shorter times (24 h). We discovered that although the differences between samples and controls are smaller, the results are less noisy.

**Figure 5.**
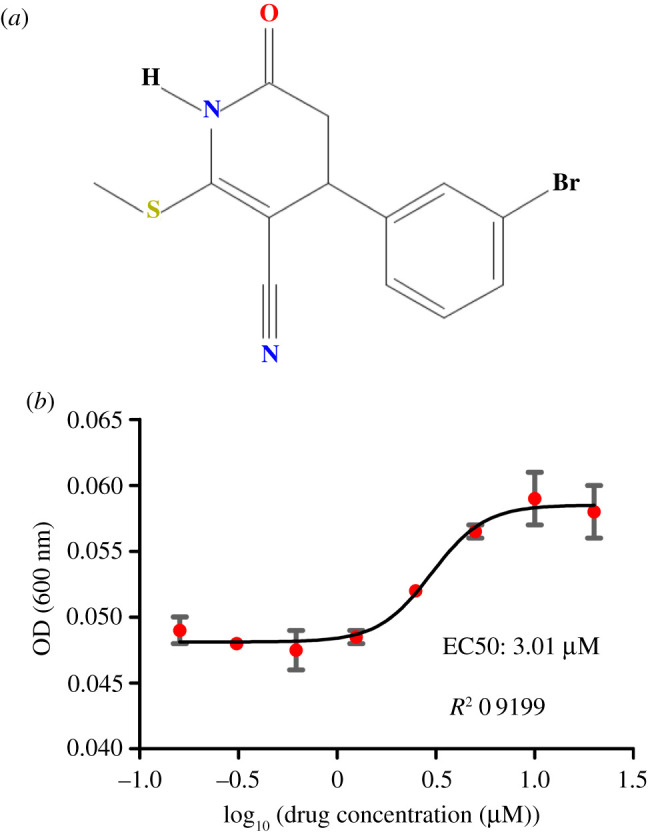
(*a*) Chemical structure for P15G3. (*b*) Dose–response curves for the JVY101 strain in the presence of P15G3 were carried out in liquid media in multiwell plates. The OD values were measured at 24 h post inoculum. Data points represent the average value for the optical densities ± standard deviation at 600 nm obtained for each concentration from two independent experiments. The data were fitted to a dose–response curve with variable slope using the software GraphPad Prism 5. The best-fit value for EC50 is 3.01 µM with a 95% Confidence Interval ranging from 2.167 to 4.172 µM. Goodness of fit: *R*^2^ 0,9199.

The only compound that had a reproducible (albeit modest) effect on the growth of JVY101 and not JVY104 is P15G3, PubChem CID 2 998 983 (4-(3-bromophenyl)-2-(methylthio)-6-oxo-1,4,5,6-tetrahydropyridine-3-carbonitrile (see figure 5*a* for the chemical structure). Figure 5*b* shows a dose–response curve for this compound, which has an EC50 value of 3 µM (with a 95% confidence interval ranging from 2.16 to 4.17 µM).

## 3. Discussion

Here we describe a yeast-based method to identify protein-palmitoylation inhibitors *in vivo*. This method is of positive selection, therefore drugs that are very toxic to the cells or have permeability issues will not be detected. The assay is simple and inexpensive compared to systems that use mammalian cells in culture. Additionally, we show that the system can be used to screen for inhibitors of at least two mammalian PATs, DHHC20 and DHHC21. Specific inhibitors for these PATs might have therapeutic potential.

We also tested a PAT gl6733 from the parasite *G. lamblia* and showed that this system can be used for the isolation of inhibitors of this PAT. While the particular role of gl6733 in the parasite life cycle has not been addressed, this strategy could be applied to target PATs which may be important for the infectivity of medically relevant parasites [58] (reviewed in [59]). Other PATs such as DHHC6 and DHHC4 did not show activity in the assay. This may be due to multiple reasons including poor expression levels, lack of activity towards Yck2, or the requirement from additional factors present in mammalian cells that are required for their activity. Indeed, not all mammalian PATs are expected to work in this system. It has been shown that some PATs are regulated by palmitoylation, generating cascades that are similar to those found in protein phosphorylation. For instance, DHHC6 requires palmitoylation by DHHC16 to be active [60], presumably this PAT would be inactive when expressed in yeast and that is what we observe in our assays.

One question that arises is why Yck2 can be palmitoylated by many PATs. The specificity of PATs is not completely understood. It has been shown in yeast that some PATs have overlapping specificities while others seem very specific for a certain type of substrate [61,62]. In particular, Akr1 activity can be complemented by overexpression of multiple PATs both from yeast and mammalian origin [53,63], indicating that Yck2 is a rather promiscuous substrate, at least in the presence of overexpressed PATs, which in this case works in our favour.

For this system to work, it is crucial that the chosen palmitoylation substrate is firmly anchored to the membrane when palmitoylated and soluble when is not. This precludes the use of substrates that might have other determinants for membrane localization such as prenylation, polybasic clusters and of course TMD.

Also, it is important that the chosen substrates are efficiently palmitoylated in yeast by the PAT we wish to inhibit, although, to some extent, this can be circumvented by the addition of 3AT.

We made some attempts to use heterologous protein domains fused to Tf, with mixed results. This strategy would benefit from a careful choice of palmitoylation substrates. As we learn more about palmitoylation determinants and PATs specificity we might be able to make more informed choices of domains that we might use to fuse to Tf, for future screens. Also, the availability of strains that have multiple yeast PATs deleted might improve our chances to run a screen using heterologous PAT and substrate pairs.

Owing to the nature of this screening method, the following compounds are expected to be found:

(I) Molecules that specifically inhibit the PAT used in the screen.
(II) Molecules that inhibit all or several PATs. These inhibitors will probably affect the mechanism of S-acylation conserved in all PATs, for example, the auto-acylation prior to palmitate transfer, or for instance, the interaction of the PaCCT motif, which is present in most PATs [64] with the catalytic domain of PATs as shown in DHHC20 structure [65]. These inhibitors, which might affect S-acylation in general, but not other cellular pathways would also be of great interest in basic research. In principle, an inhibitor that strongly affects all PATs should not be isolated by this method, since it should be lethal to yeast. However*, S. cerevisiae* can survive with up to 5 of its 7 PATs deleted [63]; therefore, it cannot be ruled out that an inhibitor that affects several, but not all PATs, or that affects all, but it is present in a sub-optimal concentration, might be found. The specificity of the inhibitors can be tested very simply, for instance by testing Akr1 inhibitors in the strains that express DHHC20, DHHC21 or gl6733. Additionally, quick and simple phenotypic tests can assess the activity of endogenous PATs, such as Swf1, Pfa4 and Pfa3 [62,64,66].

In the same line, it is expected that once the first screen with Akr1 is finalized, all subsequent screens will only focus on the hits that did not appear on the first screen, and are therefore more likely to be specific.

(III) Inhibitors that affect S-acylation indirectly. These inhibitors are also of interest from the basic point of view since they could reveal mechanisms for the regulation of S-acylation, on which there is very little information.
(IV) Compounds that interfere with the level of expression of the PATs. The decrease in the expression of a PAT could be confused with an inhibition. To rule out this possibility, PATs can be expressed from plasmids with alternative promoters. Also, PAT protein levels should be analysed by Western Blot to investigate possible post-transcriptional regulations.
(V) False positives that allow growth in the absence of histidine were the most abundant hits in our proof-of-concept screen, but they were efficiently ruled out by our counter-screen using the JVY104 strain. One such compound we identified as having histidine is its structure (P1E4); it is unclear why the others allow growth without histidine, but it is possible that they interfere with the HIS3 gene expression.

Finally, the proof-of-concept screen to find inhibitors of Yck2 palmitoylation, produced an optimal hit rate, after primary and secondary validations we obtained a compound that shows a dose–response in the micro-molar range. Further studies are being conducted to characterize the specificity and mechanism of action of this molecule. The screen has shown its feasibility and now we are focusing on finding inhibitors of DHHC20. We expect that this approach will yield the specific palmitoylation inhibitors that are much needed to advance the field of protein palmitoylation in general and to explore the therapeutic opportunities that might arise from interfering with protein palmitoylation in particular.

## 4. Material and methods

### 4.1. Strains and plasmids

NDY1949 *Mat a his3-Δ1 ura3-Δ0 leu2-Δ0 can1::STE2pr-LEU2 lyp1Δ cyh2Δ leu2Δ::LexA-HIS3::NAT-R met15Δ::YCK2(CCIIS)*.

NDY1953 *Mat a his3-Δ1 ura3-Δ0 leu2-Δ0 can1::STE2pr-LEU2 lyp1Δ cyh2Δ leu2Δ::LexA-HIS3::NAT-R met15Δ::YCK2(CCIIS) akr1Δ::LEU2*.

To generate the Tf-Yck2 expression cassette, the hygromycin-resistance gene from pFA6-hphNT1 [67] was digested using the *Not*I enzyme and cloned into the same site of pND3544 plasmid (kindly provided by Dr Nicholas Davis). This plasmid contains the YCK2pr-LexA/VP16-FLAG/HA-YCK2. The *Not*I site is upstream of the Yck2 promoter region. The whole fragment containing the hygromycin-resistance gene and Tf-Yck2 was amplified using oligos Erg6-KO-Hygro-fw and Erg6-KO-Hygro-TermYCK2-rv which bear homology to the promoter and terminator regions of ERG6. NYD1949 and 1953 were transformed with these fragments thus generating strains JVY101 and JVY102 respectively. Deletion of the ERG6 open reading frame (ORF) was verified by PCR using oligos Erg6 01, Erg6 02 and jw188. To generate the Tf-NCX1.1 intracellular loop expression cassette, the oligos α-Helix NCX1.1-fw and α-Helix NCX1.1-rev were hybridized, digested using *Xho*I and *Pst*I and cloned into pJV754, which contains LexA/VP16-FLAG/HA. The hygromycin-resistance gene from pFA6-hphNT1 was amplified using oligos oHygro-LexA-PJV42-fw and oHygro-LexA-PJV42-Rv, digested using *Xba*I and cloned into the same site in the plasmid containing LexA/VP16-FLAG/HA-NCX1.1. The whole cassette containing the hygromycin-resistance gene and LexA/VP16-FLAG/HA-α-Helix NCX1.1 was amplified using oligos Erg6-KO-Hygro-fw and Erg6-KO-Sustr-rv. NDY1953 was transformed with this fragment generating strain JVY103. Deletion of the ERG6 ORF was verified by PCR using oligos Erg6 01, Erg6 02 and jw188 (see electronic supplementary material, table S1 for a list of oligonucleotide sequences).

JVY104 was generated by deleting the ERG6 ORF on NYD1949. The hygromycin-resistance gene from pFA6-hphNT1 [67] was amplified using oligos Erg6-KO-Hygro-fw and Erg6-KO-Hygro-rv and transformed into NYD1949, hygromycin resistant strains were isolated and the deletion was verified by PCR using oligos Erg6 01, Erg6 02 and jw188.

For the expression of heterologous PATs in yeast, a Yeplac195 plasmid was modified as follows: The URA3 marker gene was replaced by MET15 by gap-repair. A TPI promoter and PGK1 terminator sequences were cloned in the polylinker to generate plasmid pJV775.

Mouse DHHC21 cDNA was amplified from DHHC21 pEF-Bos-HA vector [68] using oligos oDHHC21 01 and oDHHC21 02 and was then cloned in the *Bam*HI-*Hin*dIII sites of pJV775.

Human DHHC20 in pUC57 (optimized for yeast expression) was synthesized by GeneScript (NJ, USA), the ORF was then digested using *Kpn*I and *Pst*I and cloned in pJV775.

The *G. lamblia* PATs cDNAs (gift from Dr Andrea Ropolo) were cloned in both centromeric and episomal yeast expression vectors under the control of the constitutive TPI1 promoter. The cDNAs were cloned in both vectors using the same restriction enzymes. For GL50803_8711, GL50803_1908, EAA36893, GL50803_6733 and GL50803_2116 we used *Bam*HI and *Hind*III, for GL50803_8619 and GL50803_16928 we used *Bam*HI and *Pst*I, for GL50803_96562 we used *Xba*I and *Hind*III and for GL50803_9529 we used *Xba*I and *Sbf*I. The fragments were cloned on vectors PJV29 and PJV775 using the same sites, except for GL50803_9529 that was introduced using *Xba*I and *Pst*I.

### 4.2. Acyl-biotin exchange assays

For the detection of palmitoylated Yck2, 30 OD units of yeast cells at OD = 1 were lysed by glass bead disruption in 600 µl ABE lysis buffer (LB) (50 mM Tris buffer pH7.4, 5 mM EDTA, 150 mM NaCl and 10 mm N-ethylmaleimide (NEM)). The lysate was centrifuged for 4 min at 300 *g*, and Triton X-100 was added to 1.7% final concentration to the supernatant. The samples were incubated with rotation for 1 h at 4°C. The proteins were precipitated with chloroform:methanol. The pellets were air-dried and resuspended in 30 µl of buffer SB (4% SDS, 50 mm Tris–HCl pH 7.4, 5 mm EDTA, 10 mm NEM). In total, 120 µl of LB with 1 mm NEM were added, and the samples were incubated overnight at 4°C. The rest of the protocol was performed exactly as described in [61] but using the whole amount of protein for each sample.

Tf-Yck2 was detected using an anti-FLAG monoclonal antibody (Sigma, St Louis, Missouri, USA, product code F3165). Anti Vac8 polyclonal antibody was a gift from Dr Christian Ungermann. The blots were probed using secondary antibodies IRdye680 Goat anti-rabbit LI-COR. Product code: 926-68071 or IRdye800 Goat anti-mouse immunoglobulin G (IgG). LiCor. Product code 926-32210 (Licor Biosciences) at a 1/20 000 dilution and then scanned using an Odyssey IR imager (Licor Biosciences).

### 4.3. Yeast immunofluorescence and microscopy

IF was carried out as described in [69]. Tf-Yck2 was detected with mouse monoclonal anti-HA (1/400) (Sigma, St Louis, Missouri, USA, product code H9658) and mouse IgG Alexa Fluor 488 (Molecular Probes, 1 : 1200, product code A11029). Finally, the coverslips were incubated with Hoechst Stain 1 mg ml^−1^ for 5 min at room temperature. Yeast cells were observed in an Olympus FluoView FV1000 confocal microscope.

### 4.4. Proof-of-concept screen

96-well plates with 200 µl of SC medium without histidine were inoculated from a saturated culture, with strain JVY101 at a concentration of 0.005 OD/ml (600 nm). Subsequently, the drugs were provided at a concentration of 10 µM in DMSO. For each plate, eight wells were used as a negative control with DMSO, the drug vehicle. As a positive control, we used eight wells with strain JVY102 with DMSO. Per experiment, 10 plates are inoculated allowing the analysis of 800 compounds. The filling of the plates and the inoculum of the drugs are carried out assisted by an automated liquid handling platform (ViaFlo Assist, Integra Bioscience Corp., USA). Plates were incubated at 30°C without agitation. Cells were re-suspended by vigorous shaking for 3 min in an orbital mini shaker and the OD was monitored at 600 nm at 24, 48 and 72 h post inoculum. (EPOCH, BioTek Instruments, USA). The OD data are analysed and those compounds that produce a greater than five times the standard deviation of the negative controls were selected as Hits.

## Data accessibility

The data are provided in electronic supplementary material [70].
